# Automated design of hammerhead ribozymes and validation by targeting the PABPN1 gene transcript

**DOI:** 10.1093/nar/gkv1111

**Published:** 2015-11-02

**Authors:** Nawwaf Kharma, Luc Varin, Aida Abu-Baker, Jonathan Ouellet, Sabrine Najeh, Mohammad-Reza Ehdaeivand, Gabriel Belmonte, Anas Ambri, Guy Rouleau, Jonathan Perreault

**Affiliations:** 1 Electrical & Computer Eng. Dept., Concordia University, 1455 boul. de Maisonneuve O., Montreal, QC, H3G 1M8, Canada; 2 Biology Department, Concordia University, 7141 rue Sherbrooke O., Montreal, QC, H4B 1R6, Canada; 3 Montreal Neurological Hospital and Institute, 3801 University Street, Montreal, QC, H3A 2B4, Canada; 4 INRS - Institut Armand-Frappier, 531 boulevard des Prairies, Laval, QC, H7V 1B7, Canada

## Abstract

We present a new publicly accessible web-service, RiboSoft, which implements a comprehensive hammerhead ribozyme design procedure. It accepts as input a target sequence (and some design parameters) then generates a set of ranked hammerhead ribozymes, which target the input sequence. This paper describes the implemented procedure, which takes into consideration multiple objectives leading to a multi-objective ranking of the computer-generated ribozymes. Many ribozymes were assayed and validated, including four ribozymes targeting the transcript of a disease-causing gene (a mutant version of PABPN1). These four ribozymes were successfully tested *in vitro* and *in vivo*, for their ability to cleave the targeted transcript. The wet-lab positive results of the test are presented here demonstrating the real-world potential of both hammerhead ribozymes and RiboSoft. RiboSoft is freely available at the website http://ribosoft.fungalgenomics.ca/ribosoft/.

## INTRODUCTION

There are many uses for hammerhead ribozymes (hhRz), as they are short catalytic RNA molecules which anneal to and cleave other RNA molecules—typically transcripts of genes—leading to the knockdown of gene expression. There are other means of gene suppression via transcript cleavage, and they include the widely used RNAi (1). RNAi is effective at low concentrations, can be delivered via multiple pathways, allow for tissue specific expression and are generally accepted to be highly specific and non-toxic (2). Nevertheless, ribozymes (3), including the hhRz (the focus of this paper) (4), have relatively simple catalytic domains and can be used to target specifically RNA. They can potentially discriminate between single-base polymorphisms, target introns and sub-cellular compartments, do not require helper proteins to function and hence, can be tested *in vitro* (2). They are also easily modifiable and have been modified in ways that allow for more efficient (5,6), more specific (7,8) as well as allosteric applications involving RNA (9), and small molecules (10) or protein triggers (11). Finally, ribozymes can work in organisms that lack the typical RNAi mechanism found in eukaryotes, such as bacteria (12) or even in simple solutions *in vitro*. For these reasons, hhRz are seen as general and malleable means of transcript modification and gene therapy (13), with applications in biosensors (10) and synthetic biology as well (14).

**Figure 1.**
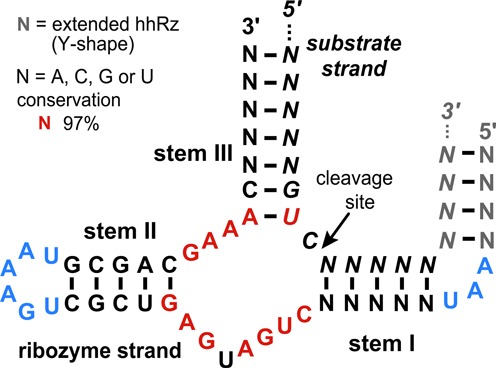
Model of trans-acting hammerhead ribozyme used by RiboSoft. Nucleotides in red correspond to the conserved catalytic core, other nucleotides may vary. Nucleotides in blue are important for the tertiary interaction between stems I and II. The sequence of stem II pictured here corresponds to the optional extended hhRz model (39) used by RiboSoft and includes the extra nucleotides in gray as well.

The hhRz is one of several structural motifs of ribozymes that can cause both cis- and trans-cleavage of RNA molecules with certain properties (3). The model of the hhRz, consists of three largely independent parts: stem I, stem II (including the catalytic core) and stem III. A trans-cleaving ribozyme annealed to a target substrate strand is shown in Figure 1. Stems I and III are chosen to be complementary to the substrate strand, which must include an NUH triplet. Stem II is found next to the catalytic core's conserved GAAA and CUGANGA. Generally speaking, a designer would pick an empirically proven stem II and catalytic core, then vary the two ‘arms’ (call them I and III) so they would form stems I and III, with pre-specified characteristics, with the targeted substrate.

Both target specificity and turnover (re-use) rate of a hhRz depend on the length and sequence of its two arms. Optimal turnover requires that product dissociation not be slow. For long substrates with extensive secondary structure, such is the case with most mRNAs, the optimal flanking arms may be longer than 10 nt, but even in this case, increasing the arms too much reduces turnaround rate greatly.

This paper introduces a web-service that makes it easy for interested parties—not only ribozyme experts—to (i) design hhRz that target a particular gene, or any RNA sequence, and (ii) make an informed choice as to which of the possible ribozymes are best for a particular application. All this while automatically taking in account the multiple variables that determine ribozyme efficacy (target structure, ribozyme-target annealing and ribozyme structure, including the tertiary interaction of so-called extended ribozymes). In addition, we have used this tool to design hhRz, which were tested for efficient cleavage of three transcripts, as well as *in vivo* for the PABPN1 gene, the root cause of OPMD.

OPMD (Oculopharyngeal muscular dystrophy) is a late onset (around 50 years old) muscle disease with occasional involvement of the nervous system that can lead to obligatory wheelchair usage (15,16). Genetically, diagnosis requires detection of an expansion of a GCN trinucleotide repeat in the first exon of PABPN1. Normal alleles contain 10 GCN trinucleotide repeats; autosomal dominant alleles range in size from 12 to 17 GCN repeats; autosomal recessive alleles comprise 11 GCN repeats (16).

Spinocerebellar ataxia type 3 (SCA3), like OPMD, is a trinucleotide repeat neurodegenerative disease caused by the polyglutamine (versus polyalanine) expansion in the deubiquitinating enzyme, Ataxin-3 (*ATXN3*). Using the RNA interference mechanism, Rodríguez-Lebrón, E *et al*. made microRNA mimics that targeted the 3′-untranslated region of a human *ATXN3* expressed in the cerebellum of transgenic mice (17). This resulted in silencing of the human gene and cleared the abnormal nuclear accumulation of the mutant protein. Most OPMD-afflicted patients have a wild type PABPN1 allele and a mutant (disease-causing) PABPN1 allele, so targeting the 3′-UTR of the mRNA of PABPN1 (with microRNAs or ribozymes) will suppress both alleles. Hu *et al*. (18) demonstrated that the targeting of the trinucleotide repeats can *preferentially* inhibit mutant *ATXN3*. However, an ideal solution is one that would *exclusively* impact the mutant allele or *compensate* for the knockdown effect on the wild type allele. This could be achieved by cleaving both alleles with a hhRz and providing a replacement mRNA with sequence modification rendering it immune to cleavage. HhRz were shown to efficiently suppress beta-secretase gene expression in HEK293 (human embryonic kidney) cells (19). In the sequel, we show that RiboSoft can generate the designs of active hhRz that were proven effective *in vitro* and *in vivo* against PABPN1 sequence. We also tested a dozen ribozymes that proved capable of cleaving RFP or a 16S rRNA fragment *in vitro*. Finally, we show that the combined use of ribozymes can achieve near complete depletion of the target RNAs.

## MATERIALS AND METHODS

### RiboSoft usage

**Figure 2.**
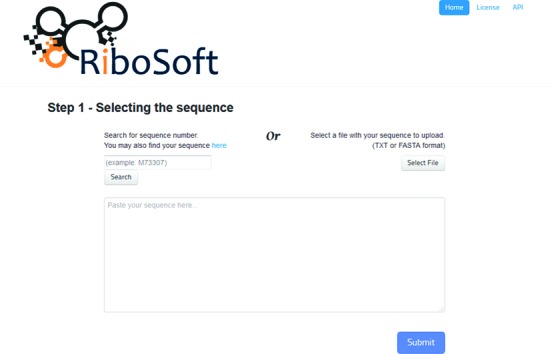
Snapshot of the input screen (step 1) of RiboSoft: asking the user for a sequence, a file or a sequence code.

**Figure 3.**
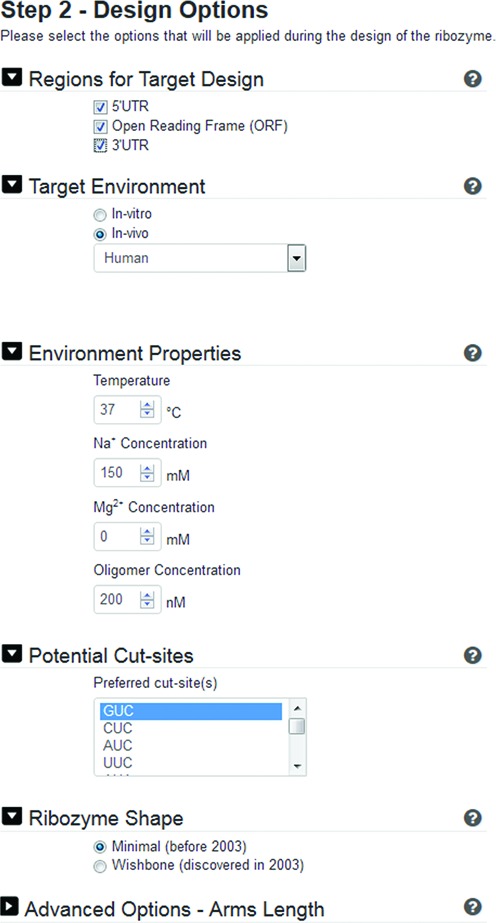
Partial snapshot of the input screen (step 2) for RiboSoft: allowing for user specification of various parameters.

Before the design algorithm can run, valid input from the user must be entered. The required input takes the form of (i) a target nucleotide sequence, which may be provided by: direct entry into a text box, uploading a pure text (.txt) or FASTA format file, or typing in a GenBank sequence number (Figure 2); and (ii) a set of design parameter values (Figure 3).

**Figure 4.**
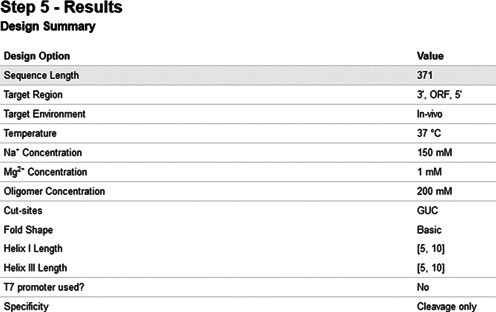
Snapshot of the input screen (step 3) for RiboSoft: confirmation of user input.

The design parameters are: (i) the target environment: whether it is *in vitro* or *in vivo*, and if it is *in vivo*, then the name or code of the intended organism; (ii) environment properties: temperature as well as oligomer, sodium and magnesium ion concentrations; (iii) potential cut-sites (e.g. GUC); (iv) ribozyme structure: minimal or extended (i.e. involving loop-loop interaction, designated as wishbone-shaped) (5); (v) advanced options: the minimum and maximum lengths of helices I and III (the targeting arms of the ribozyme), the presence or absence of a T7 promoter (usually used for *in vitro* experiments) and whether specificity should take into consideration off-target sites that allow annealing only or annealing and cleavage by the designed ribozyme. After the parameters are chosen, they are displayed on a summary page (Figure 4) before requesting the user's email to submit the ribozyme design request.

**Figure 5.**
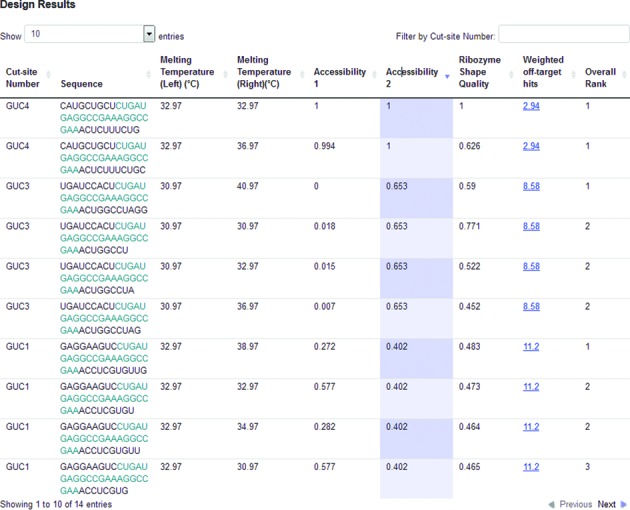
Sample output from RiboSoft. The cut-site number (starting at 0 for the first cut-site) and corresponding ribozyme sequence is shown for each candidate. All the parameters corresponding to this candidate are shown on the same row, Tm of both arms, accessibility (from 0, worst, to 1, the best), ribozyme shape (also from 0 to 1) and the overall rank. Off-target hits are shown only when the ribozyme was selected for *in vivo* use, 1 normally means that only the targeted gene is hit, higher numbers imply potential non-specific activity.

A target sequence must be provided, but most of the design parameters have default values, which are acceptable for most applications. The design algorithm then initiates the actual design and optimization procedure. This procedure has six steps, which starts with the generation of a large number of candidate ribozymes, assessing them using various measures of quality, including (but not limited to) target cut-site accessibility and specificity to the target sequence as well as proper folding of the ribozyme structure. The procedure concludes with the multi-objective assessment and ranking of resulting candidate ribozymes (Figure 5). The algorithms for each part of the procedure are explained below.

### Candidate generation

In principle, a hhRz can cleave, with different efficiency levels (measured by different studies with varying results [20,21]), at locations on RNA strands with an NUH motif. An RNA strand may have any number of different cut-sites within it, repeated any number of times. The most widely targeted cut-site is GUC, as it is thought to be the most susceptible to hhRz cleavage. For all cut-sites, all possible candidates with perfectly hybridizing arms, of lengths between the minimum and maximum values specified by the user, are generated. The total number of candidates is calculable using the formula:

(1) \begin{equation*} \approx (arm{\rm - }length\,range)^2 \cdot (number\,of\,cut{\rm - }sites) \end{equation*}

This implies that the number of candidates is also proportional to the target sequence length, since more cut-sites will be found. To provide a rough estimate of the number of candidates that are typically generated by RiboSoft, with a conservative length of 3–10 for the hybridizing arms and two cut-site types, a sequence of 3000 nucleotides yields about 100 cut-sites for two cut-site types for a total of 6400 ribozyme candidates.

### Annealing temperature

A trans-cleaving hhRz may be viewed as a three-part construct: a fixed largely double-stranded central segment (core and stem II), plus two single-stranded arms (stems I and III) up- and down-stream of the core. It is those two arms that provide the selectivity that permit the ribozyme to strongly anneal to a particular site on a target RNA molecule amongst myriad RNA strands in the medium and hence, cleave that target specifically.

The shorter an arm is, the lower the degree of selectivity and strength of annealing (annealing temperature) between the arm and the target RNA. In converse, the longer an arm is the more selective the annealing and the more heat is required to de-anneal the arm from the target RNA. Thus, it may appear that the solution is to automatically give every hhRz very long arms, but the catch is that longer single-stranded arms make it more likely that an arm would anneal to itself and/or with the other arm (partially or fully). Also, ribozymes with very long arms will anneal so strongly to their target that they will not turnover to anneal to new targets after cleavage.

In brief, the annealing temperature of a hhRz may be approximated by the sum of the annealing temperatures of the two arms of the ribozyme. All environmental conditions (e.g. ionic concentrations) being equal, the annealing temperature is a function of the nucleotide make-up and lengths of the arms of a ribozyme. A number of formulas have been used and compared (22,23) to calculate the annealing temperature of RNA (and DNA) molecules, including the fairly standard base-stacking approach employed by our algorithm (adapted from http://www.basic.northwestern.edu/biotools/oligocalc.html (24)).

Since calculation of the annealing temperature is computationally simple (relative to RNA folding) we use this calculation in a discriminatory manner to weed out all candidate hhRz with arms that are too short or too long. In fact, the range of acceptable annealing temperature we use is from –5°C to 60°C above the temperature of the intended environment for each for each arm separately. It is acceptable for a single arm to have a low Tm if the other arm makes up for it with a highly above average Tm. The exact values were determined by empirically testing many ranges to avoid eliminating perfectly sound sequences.

For a given cut-site on a target RNA strand, the sequence of any arm of a hhRz is dictated by the RNA sequence around the cut-site, as a hhRz should anneal perfectly to its target. Hence, every hhRz with target-matching arms and an overall annealing temperature within the pre-specified range is an acceptable hhRz candidate generated by this phase of the design process.

### Ribozyme structure

Every hhRz candidate surviving the annealing temperature phase is evaluated for its structure. This is a measure of how open (single-stranded) the arms are, which makes it easier for a ribozyme to find and anneal to its intended target sequence and hence, perform its trans-cleavage function. The Sfold program (25) is used on the server side to fold all the candidates; it uses a statistical Boltzmann ensemble model to generate possible secondary structures for the candidate RNAs. Sfold reports 10 secondary structures, each with its own probability. This probability is used as a weight when considering the structure of a ribozyme.

RiboSoft gets the quality of one or many secondary structures for one hhRz with an algorithm that uses the sum of the melting temperatures of all continuous stretches of double-stranded RNA as an (inverse) measure of the openness of the two arms of the ribozyme. We allow up to one mismatch in each ‘continuous’ stretch of double-stranded RNA. All of the temperature values used are in Kelvin, as cancelation may erroneously occur with negative temperatures. A pseudo-code description can be found in supplementary information.

For each secondary structure of the 10 secondary structures associated with a given hhRz, we compute one overall melting temperature. Naturally, continuous pairs in the hhRz (Helix-II) will not be counted, since they are meant to be there. However, a penalty of 50 degrees is added to any structure lacking the hammerhead core, i.e. where the conserved bases of the core are base paired or where Helix-II is not formed. Since the resultant temperature here is associated with only one of 10 possible structures, a weighted sum (*Q*_f_) of all 10 temperatures is calculated, for each hhRz candidate, using the formula:

(2) \begin{equation*} Q_f = \sum\nolimits_{i \in \bf{Q}} {f_s \cdot Q_s } \end{equation*}

where *Q*_s_ is the melting temperature of one secondary structure; *f*_s_ is the probability associated with that secondary structure; **Q** is the set of all 10 structures. Naturally, lower values for *Q*_f_ are preferred and as such, this measure of quality should be minimized.

### Target accessibility

Accessibility is a measure of how easy it is to access the target. Targets that fold unto themselves strongly in the region around and including a cut-site will reduce the efficiency of a ribozyme designed for that cut-site.

Two accessibility terms are computed here. One term, *cut-site inaccessibility*, inversely measures the openness of a target cut-site, to a particular hhRz candidate with specific arms’ lengths. This is reflected by the annealing temperature of the region of the target cut-site, the region to which the arms of a hhRz must anneal. The other term, *disruption energy* (Δ*G*_disruption_) is based on research done by (26), and is in fact: the free energy cost to melt any local secondary structure at the target site, making it accessible to hhRz binding.

The computation of cut-site inaccessibility is done per candidate hhRz, as different ribozymes targeting the same cut-site will have different arms’ lengths. The substrate is also folded using Sfold, which returns 10 secondary structures for the cut-site region, hence 10 annealing temperature values are computed for that region, and these are summed using the probabilities of the corresponding secondary structures as weights. The result of this calculation is the raw cut-site accessibility value (both *cut-site inaccessibility* and *disruption energy* are succinctly described in *pseudo-code*, supplementary information).

The calculation of disruption energy is done per cut-site. The reason for this is the significant computational cost associated with the computation of the secondary structure for a long RNA strand; typical gene transcripts are one to several thousand bases long. We use 23 bases for both arms’ binding sites and hence compute Δ*G*_disruption_. This allows the evaluation of the structures likely to compete most strongly with hhRz binding. Since Sfold returns 10 possible secondary structures for the target transcript, we will also have 10 Δ*G*_disruption_ values. These values are used to calculate a weighted linear sum in a fashion identical to what was done for cut-site accessibility.

As with ribozyme structure, cut-site inaccessibility and disruption energy should be minimized. In other words, RiboSoft looks both at the ease with which the ribozyme can bind to the target site, as well as the structure of hhRz and target.

### Specificity assessment

For *in vivo* applications it is important that a given ribozyme affects the targeted transcript and no other. Typically, a hhRz would affect a transcript by cleaving it. It is possible, however, for a hhRz to anneal to a transcript and hence, lead to the transcript's destruction or to a reduction in its translational efficiency, without cleaving it. This is part of what is known as off-target effects. Hence, the ‘advanced options’ section of RiboSoft's input pages allow the user to check for either off-target hits that would potentially result in cleavage or *all* off-target hits, including those that do not lead to cleavage.

The NCBI BLAST web-service is queried with a high threshold (sensitivity) using the region around the targeted cut-site corresponding to the maximal possible lengths of hhRz arms. The results are weighted based on the number of matched pairs, thus a perfect match yields a weight of one (the targeted transcript). It should be noted that RNAs with accession numbers starting with XM or XR (RNA predicted sequences) are ignored in the count, but are still reported in the results’ page.

The program will only return 0 if the RNA sequence does not exist, since there is not necessarily *a priori* knowledge of the transcript being targeted (users can enter arbitrary sequences directly). Optimally, the value of specificity is 1, entailing a perfect match to only one cut-site on the targeted transcript. Higher specificity values are detrimental to the quality of the hhRz candidate, and as such this measure of quality should be minimized (*pseudo-code* in supplementary information briefly describes this).

### Hammerhead ribozymes

The hammerhead DNA templates, recommended by RiboSoft for PABPN1, were prepared by 5 cycles of polymerase chain reaction (PCR) using Taq DNA polymerase and 1 μM of the T7 promoter 5′-TAATACGACTCACTATAGCG-3′ with 1 μM of each oligonucleotide (sequences complementary to the T7 promoter are underlined):

5′-GGCCTGGAGTTTCGTCGCATTTCAGCGACTCATCAGTGAGGTTAAACTGGAGCCTGCGCTATAGTGAGTCGTATTA-3′, 5′-TATCAAAGCTCGAGTTTCGTCGCATTTCAGCGACTCATCAGAGGGATTAGATGGAGGAAGACGCTATAGTGAGTCGTATTA-3′, 5′-AGATGAATATGAGTTTCGTCGCATTTCAGCGACTCATCAGCACCTTTACCAGGCAATGCGCTATAGTGAGTCGTATTA-3′, 5′-GGGTCGCGTTTCGTCGCATTTCAGCGACTCATCAGTACAGTTAGGGCCGGCGCTATAGTGAGTCGTATTA-3′. corresponding respectively to the constructions for Yz144, Yz363, Yz437 and Yz867, with an annealing temperature of 55°C with 200 μM dNTP, 2 mM MgCl_2_, 120 mM Tris–HCl pH 8.8, 10 mM KCl, 10 mM (NH_4_)_2_SO_4_ and 0.1% Triton X-100. The sizes of the DNA templates were confirmed on 1.5% agarose gel. Full annotated ribozyme sequences, as well as the target RNAs, are available in supplementary materials.

### PABPN1 gene

The PABPN1 gene used to create by PCR the DNA template for transcription was the D323 clone used in (16). The first PCR product was made with the oligonucleotide 5′-GACTACGGGAACGGCCTGGAGTC-3′ starting 129 nucleotides downstream of the start codon and with the oligonucleotide 5′-AAAGGGAACAAAAGCTGGAG-3′ 147 nucleotides downstream of the stop codon (as reverse complement); covering 936 nucleotides. To generate a full-length PCR product, 20% betaine was incorporated to the PCR reaction (same reaction conditions as above) for 30 cycles at an annealing temperature of 51°C. A second PCR reaction was performed on the first PCR product with identical conditions, except for the first oligonucleotide being replaced by the oligonucleotide 5′-TAATACGACTCACTATAGCGACTACGGGAACGGCCTGGAGTC-3′ to incorporate the T7 promoter to the DNA template used for transcription. The size of both DNA templates were confirmed on 0.7% agarose gel. The PCR product was purified with an EZ-10 Spin Column PCR Products Purification Kit (Bio Basic Inc.) and precipitated in ethanol with NaOAc.

### RNA synthesis of hammerhead ribozymes

Ribozymes were synthesized by *in vitro* run-off transcription in 100 μl reactions using 20 pmol of double-stranded DNA template, 80 mM HEPES–KOH pH 7.5, 24 mM MgCl_2_, 2 mM spermidine, 40 mM DTT, 3.0 mM of each NTP, 0.01 U of yeast pyrophosphatase and 2 μg of purified T7 RNA polymerase at 37°C for 3 h (27). The reactions were stopped by adding 5 U of DNase (RNase free) and incubating at 37°C for 30 min. The mixtures were extracted twice with both phenol and chloroform, and the nucleic acid precipitated with ethanol. After dissolution in equal volumes of water and formamide dye buffer (95% formamide, 10 mM EDTA, 0.025% bromophenol blue and 0.025% xylene cyanol) the ribozymes were fractionated by 10% denaturing (8 M urea) polyacrylamide gel electrophoresis (PAGE; 19:1 ratio of acrylamide to bisacrylamide) in buffer containing 45 mM Tris-borate pH 7.5 and 1 mM EDTA. The reaction products were visualized by UV shadowing and the bands corresponding to the ribozymes cut out. The transcripts were eluted from the gel slices overnight at room temperature in a solution containing 0.1% SDS, 10 mM EDTA and 0.5 M ammonium acetate. The purified RNA was precipitated by the addition of 0.1 volume of 2 M sodium acetate pH 4.5 and 2.5 volumes of ethanol. After washing in 70% ethanol and drying, the pellets were resuspended in ultrapure water, and the quantity of RNA determined by spectrophotometry at 260 nm.

### RNA synthesis of PABPN1 gene

The full-length PABPN1 gene could not be transcribed efficiently. Various conditions and constructions were tested until a construction with a truncated 5′-end was successfully transcribed at a reasonable amount. The ‘standard’ transcription protocol described for the ribozymes was however inefficient for this gene. Transcripts were obtained only with the HiScribe™ T7 *in vitro* Transcription Kit (New England Biolabs) where 50 pmol of the column-purified and precipitated DNA template was in presence of 20% betaine and the kit's supplied buffer, High Molecular Weight solution, rNTPs and T7 RNA polymerase enzyme. The transcription reaction proceeded for 4h30 at 42°C and was stopped by the addition of 5 U of DNase (RNase free) and incubating at 37°C for 45 min. Followed by phenol-chloroform extraction and precipitation, the RNA was purified on 5% 8 M urea PAGE. Elution, precipitation and concentration determination were performed as stated above.

### RNA labeling

Purified PABPN1 RNA (6 pmol) was 5′ dephosphorylated with 5 U antarctic phosphatase in 50 mM Bis-Tris-Propane-HCl pH 6.0, 1 mM MgCl_2_ and 0.1 mM ZnCl_2_ for 30 min at 37°C followed by heat inactivation 5 min at 65°C. The RNA was radioactively 5′-^32^P-labeled using 10 U of T4 polynucleotide kinase (NEB) with 20 μCi of [γ-^32^P]-ATP (3000 Ci/mmol; Perkin-Elmer) for 1 h at 37°C in 70 mM Tris-HCl pH 7.6, 10 mM MgCl_2_ and 5 mM DTT. The labeled RNA was gel purified on 5% 8 M urea PAGE, eluted, precipitated as stated above except for the RNA bands being identified by radioactivity rather than UV shadowing.

### Kinetic measurements of ribozyme cleavage

Trace amount of [5′-^32^P]-radioactively labeled PABPN1 RNA and 10 nM of ribozyme (10 nM of each ribozymes when in combination) were separately pre-incubated 10 min at 37°C in the presence of cleavage buffer with ionic strength roughly similar to physiological conditions (50 mM Tris pH 7.5, 100 μM MgCl_2_, 100 mM NaCl and 25 mM KCl). The cleavage reaction was initiated by adding an equal volume of target to the ribozyme. Aliquots (1.5 μl) were removed at intervals and the reaction terminated by addition to a 5 μl chilled mixture containing 95% (v/v) formamide, 10 mM EDTA and electrophoresis dyes. Ribozyme and cleavage products were separated by electrophoresis in a dual concentration 8 M urea 19:1 polyacrylamide gel with a 4% top two-third and 20% bottom third. Gels were exposed to storage phosphor screens, and quantified by phosphorimaging. Proportion of cleavage was calculated by dividing the intensity of bands corresponding to ribozyme cleavage products, divided by the sum of all bands in the lane (including the full length RNA). Progress curves were fitted by nonlinear regression analysis to single exponential function.

### Construction of plasmids for ribozyme expression in mammalian cells

The same four hhRz against PABPN1 were used, namely Yz144, Yz363, Yz437 and Yz867. Templates containing these hammerhead catalytic sequences were synthesized chemically. The sequences of these four ribozymes are:

- Yz144: 5′ CAGGCTCCAGTTTAACCTCACTGATGAGTCGCTGAAATGCGACGAAACTCCAGGCC 3′
- Yz363: 5′ TCTTCCTCCATCTAATCCCTCTGATGAGTCGCTGAAATGCGACGAAACTCGAGCTTTGATA 3′
- Yz437: 5′ GCATTGCCTGGTAAAGGTGCTGATGAGTCGCTGAAATGCGACGAAACTCATATTCATCT 3′
- Yz867: 5′ CCGGCCCTAACTGTACTGATGAGTCGCTGAAATGCGACGAAACGCGACCC 3′

PCR products were digested with KpnI and Csp45I restriction endonucleases and were ligated with pUC-KE-tRNA-CTE plasmids that had been digested with both KpnI and Csp45I obtained kindly from Nawrot B. (19). The DNA sequence of every plasmid was verified using Sanger sequencing with the PCR primer P7 (5′-CGCCAGGGTTTTCCCAGTCACGAC-3′).

### Cell culture and transfection

Human transformed primary embryonal kidney cell lines HEK293 were cultured in Dulbecco's modification of Eagle's medium (Invitrogen), supplemented with 10% foetal bovine serum, G418 (200 μg/ml) was used as selection antibiotic for ribozyme-transfected HEK293 cells. Cells were grown in monolayer, at 37 °C in cell culture incubator with 5% CO_2_ in 6-well plates and transfected at 70–80% confluence. Transfection with 2 μg of the ribozyme containing plasmid was carried out using the Jet prime reagent (Polyplus-transfection Inc., France) according to the manufacturer's protocol. One week later, selected G418 cells were collected for RNA and protein extractions in parallel.

### Western blotting

Total proteins were extracted in SUB buffer at different time points (containing 8M urea, 2% mercaptoethanol and 0.5% SDS), and electrophoresed in 12% SDS-PAGE gels. Proteins were blotted into nitrocellulose membrane and probed with specific antibodies against PABPN1 (1:2000) (Abcam, USA). Parallel samples were probed using monoclonal actin antibody (Chemicon International, USA) to confirm the equal loading of lysates between lanes. After incubation with specific secondary HRP-conjugated antibodies, the membranes were revealed using the western blot chemiluminescence reagent plus kit (NEN Life Sciences Products, Boston, MA, USA).

Quantification of the band intensity was performed using the ImageJ software. Values were normalized by β-actin. Quantification of PABPN1 protein level was determined by western blot using the ImageJ software, normalized to actin and presented as fold change relative to the level of PABPN1 in untransfected HEK293T cells.

## RESULTS

### Fitness evaluation

The quality of a candidate ribozyme is reflected in all five terms computed in sections above. It should be noted that the raw values returned by some of those quality measures, are not intuitively comprehensible. For instance, greater values for ribozyme structure indicate worse structures. In addition, it is often not clear what the lower and upper bounds are, for a given measure. For these reasons, the following equation is used to normalize the values returned by most of the quality measures:

(3) \begin{equation*} M_{normalized} = \frac{{(M_{original} - M_{\min } )}}{{M_{\max } - M_{\min } }} \end{equation*}

where *M*_min_ is the smallest value for that measure among all candidates, and vice versa for *M*_max_. *M*_original_ is the original (raw) value for that measure, while *M*_normalized_ is that value after normalization. Also, if for a given measure larger values are detrimental, the following equation is applied to the output, so that 1 represents the best possible value and 0 the worst:

(4) \begin{equation*} M_{adjusted} = 1 - M_{normalized} \end{equation*}

*M*_normalized_ is the normalized value for that measure, while *M*_adjusted_ is that value inverted, and is the final value used by the multi-objective optimization procedure outlined below. Since melting temperature and specificity can be accurately judged by the user, they are not normalized or adjusted.

Depending on the application, the designer will have a greater interest in certain parameters versus others. For example, specificity or lack of off-target effects may be of great value to some *in vivo* applications, but would probably be of no concern to most *in vitro* applications. Hence, it is not possible to identify beforehand, and for all possible applications of a hhRz, what the relative importance of the various terms should be. Even for a particular well-defined application, it is sometimes impossible for a user to spell-out numerically the relative importance of the various quality terms. As such, we adopted a multi-objective optimization approach to the ranking of the ribozymes.

This approach (summarized in supplementary material and described in full in [28]) allows us to present the various candidates to users, as members of various Pareto optimal fronts. In other words, all ribozymes ranked 1 correspond to an optimal choice between the parameters used by RiboSoft such that choosing another candidate ribozyme with one of these parameters ‘improved’ would actually lead to a less efficient ribozyme due to other parameters being adversely affected. This lesser candidate would be ranked as 2 with other such candidates ‘only one value away from the optimum’, similarly, ribozymes ranked 3 would be less optimal than 2, and so on until all candidates not rejected by RiboSoft cut-offs are ranked. In order for the user to make a final decision as to the best hhRz for his application: both rank and the various quality measures are included in a table of all hhRz candidates. The table can be sorted, by the user, according to any of the individual measures as well as overall rank. This table constitutes the output of the web service (RiboSoft) implementing the algorithm (Figure 5). Additional details (including *pseudo-code* and examples of results) are provided in supplementary information.

### Single ribozyme cleavage and additive effect of combinations

In addition to specifying and devising a comprehensive hhRz design algorithm, then implementing it as web service, we have also used the service for a particular gene silencing application. This involved the PABPN1 gene, whose mutation results in a muscular dystrophy disease known as OPMD. Before proceeding to *in vivo* trials in various model organisms, which is a costly affair, we proceeded to test the validity of ribozymes generated by RiboSoft, *in vitro*, for their efficiency in cleaving the transcript (mRNA) of the PABPN1 gene. The following two sections describe in detail the wet lab experimental methods followed to test the ribozymes and the results of these tests. The results were positive and provide preliminary evidence of the value of the new web service.

**Figure 6.**
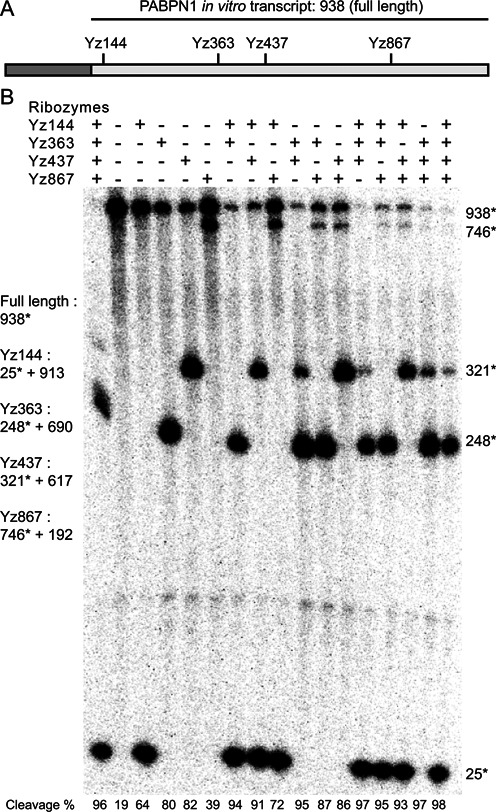
Single and combined ribozyme cleavage. (**A**) The PABPN1 transcript is illustrated. Gray box corresponds to the region that includes the mutations, but is not included in the transcript for this assay because we were unable to fully transcribe it *in vitro*. White box corresponds to the portion of the transcript used for the assay, with cleavage sites indicated. (**B**) Cleavage assay. The 938 bases substrate labeled in 5′ was incubated 40 min at 37°C, with 100 nM of the indicated ribozymes, in 50 mM Tris, pH 7.5, 100 mM NaCl and 100 μM MgCl_2_, except for lane 1, which was in 0 MgCl_2_. Reactions were loaded on a 4% (top)/ 20% (bottom) denaturing PAGE. Numbers with asterisks correspond to the labeled fragment. The efficiency of cleavage, represented as a percentage of the original un-cleaved transcript, is shown at the bottom of the figure. With the exception of the first lane, the results are arranged, from left to right to reflect an increasing number of participating ribozymes. Naturally, whatever remains of the original transcript is represented by the bands at the top of the figure, while the various fragments resulting from cleavage are spread all over the gel below. The expected lengths of the transcript fragments are listed to the left of the gel, while the actual lengths of the detected fragments are listed on the right at the proper location. This gel is a representative experiment of three assays.

The *in vitro* efficiency of cleavage by the designed hhRz of the PABPN1 transcript is shown in Figure 6. The experiment measured the efficiency of cleavage of all single, double, triple and quadruple combinations of four ribozymes. We tested the efficiency of the ribozymes in conditions similar to those often used to assay ribozymes intended for use in mammalian cells (37°C, 100 mM Na^+^ and 100 μM Mg^2+^). The only exception being the very first lane in Figure 6, where a combination of all four ribozymes was used at zero magnesium ion concentration.

The results indicate several important facts: (i) all single ribozymes cleave the PABPN1 transcript, with an efficiency of cleavage ranging between 39.3% and 81.5%. (ii) A greater number of ribozymes invariably cleaves more efficiently than a smaller number of ribozymes; the average cleavage percentage for 1, 2, 3 and 4 ribozymes is: 66.15%, 87.65%, 95.575% and 97.7%. (iii) Oddly enough, a combination of all four ribozymes in the absence of magnesium ions also cleaved very efficiently (96.6%) even if magnesium is thought to be essential for efficient cleavage by hhRz. (iv) The greatest increase in the efficiency of cleavage resulted from using two instead of one ribozyme. This result confirms that the design of the ribozymes, amongst many generated by RiboSoft, were correct, in that they led to functional ribozymes.

To ensure the universal applicability of RiboSoft, we assayed 15 additional hhRz designed by our software against two very different targets, a RFP mRNA and a portion of a 16S ribosomal RNA, the latter is notably structured and likely much less accessible than the average mRNA. All the ribozymes that were ranked 1 against these targets could cleave similarly to the results obtained for PABPN1-hhRzs, except for one inactive ribozyme, as well as most ribozymes with ranks as low as 7 (Supplementary Figures S1, S2, Table S1 and data not shown). However, for two ribozymes that had poor rankings, and interestingly had an *accessibility 2* measure of zero, no significant cleavage was observed (Supplementary Table S1). Two hhRz based on the same cut-sites, but designed manually, did not cleave either, providing additional evidence that ‘good ranking hhRz’ designed by RiboSoft warrant active ribozymes. As with PABPN1, the combination of ribozymes led to nearly complete degradation of the substrate RNA (Supplementary Figure S1 and S2).

### Single and combined ribozymes kinetic reaction results

**Figure 7.**
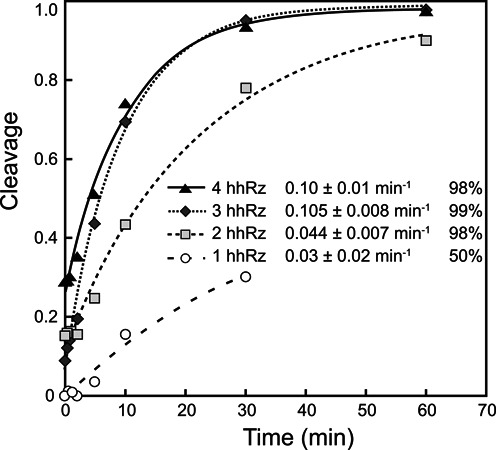
Single and combined ribozyme kinetic. The rate of cleavage of one, two, three and four combined ribozymes is illustrated. Cleavage was quantified and plotted to determine rate constants, which are indicated within the graph together with the final percentage of cleavage: open circle, RNA substrate cleaved by a single ribozyme (Yz144); by two ribozymes (Yz144 and Yz363); three ribozymes (Yz144, Yz363 and Yz437); and with all ribozymes. Nine time points are taken at 0 s, 20 s, 40 s, 1 min, 2 min, 5 min, 10 min, 30 min and 1 h. These results are representative of two assays.

The progress of the cleavage reaction over time, for all combinations of the four ribozymes, is presented in Figure 7. The purpose of these data is to see and mathematically model the rate of the cleavage reaction. This allows us to identify the time it takes the reaction to reach its steady-state. The plots provide a complete picture of the dynamic relationship between the number of ribozymes (participating in a reaction) and the rate of cleavage over time. The efficiency of cleavage at each time step can be seen in Figure 7 as well as the constants of the cleavage rate (k_obs_).

It can be seen from the plots that both the rate of the cleavage reaction and the steady-state value of cleavage efficiency have increased due to the presence of an additional ribozyme. The two-ribozyme combination was able to cleave about 43% of the transcript in 10 min, while the single ribozyme cleaved about 15% in the same time period. Also, the two ribozyme combination had a steady-state cleavage efficiency of ∼90% a clear improvement over the steady-state of the single ribozyme of ∼ 30%. Similarly, the combination of three ribozymes increased further the cleavage efficiency of the RNA substrate. Indeed, only 5 min was required to cleave 44% of the target RNA, half as much time as with two ribozymes, which achieved 43% cleavage in 10 min. In spite of this, three ribozymes presented only a minor improvement over the already highly efficient combination of two ribozymes, 90% cleavage. No measurable improvement was obtained with four ribozymes compared to three.

It is recommended that one uses two proven hhRz in gene silencing applications; three or more hhRz is not advisable, as that increases the cost of construction and delivery without considerably enhancing the steady-state value of cleavage efficiency. Also, expressing many ribozymes increases the likelihood of unwanted side-effects such as off-target hits and toxicity for *in vivo* applications.

### Specific hhRzs are able to inhibit PABPN1 expression level

**Figure 8.**
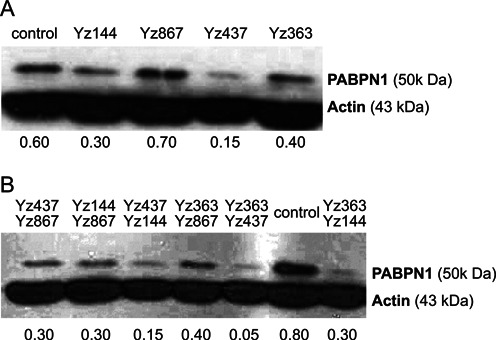
Ribozymes are effective in knocking down PABPN1. (**A**) Different individual ribozymes targeting PABPN1 transcript are able to knock down the level of PABPN1 protein. The endogenous expression of PABPN1 was analyzed by western blot. HEK 293T cells were transfected with the corresponding individual ribozymes, and selected with G418 for 5 days before protein extraction. (**B**) Combination of two ribozymes targeting PABPN1 transcript are more efficient in knocking down the level of PABPN1 protein. The endogenous expression of PABPN1 was analyzed by western blot. HEK 293 cells were transfected with the combined two indicated hhRzs. For (A) and (B), the ‘control’ lanes correspond to plasmid without ribozymes. Actin antibody was used to confirm equal loading. The values below the gel indicate PABPN1 protein signal intensities (quantified using ImageJ) after normalization to actin signal intensities and to normal PABPN1 protein levels (in cells that have not been transfected).

To test the hhRzs silencing effect, we used the human cell line HEK293T and transfected the cells with all different hhRzs individually. Stable clones of hhRzs were maintained in G418 selective media all the time during manipulations. We then performed western blot on cell extracts to test the effectiveness of hhRzs in knocking down the endogenous PABPN1 level in this cell line. Western blot results revealed hhRzs sequences: Yz144, and Yz437 as optimal candidates, as they showed high efficiency in knock-down PABPN1 (Figure 8A). We then transfected combinations of two hhRzs simultaneously in HEK293T cells and performed western blot to test if they are more effective in knocking down PABPN1 protein level. A combination of two hhRzs showed more reduction in PABPN1 protein level (Figure 8B) than individual hhRzs (Figure 8A), reducing expression down to 5% for the combinations of Yz363 with either Yz437 or Yz144. In addition to providing a strong promoter, the pUC-KE-tRNA-CTE plasmid includes a tRNA to help scaffold and export RNA to the cytoplasm, as well as the Constitutive Transport Element (CTE) which tethers an RNA helicase that helps unfold RNA and facilitates hhRz binding to its target mRNA at the same time. While concerns could arise from the use of ‘non-ribozyme’ sequence in the 5′ portion of the constructs, this extra sequence is expected to fold in a modular fashion independent from the hhRz. The excellent activity of the ribozymes against their target *in vivo* confirms that the ribozymes are still active.

## DISCUSSION

This paper presents two main contributions to the world of ribozyme design and ribozyme application. The first part of the paper describes the algorithm and implementation of RiboSoft, a completely automated program (offered as a web service) for hhRz design. It can be used to design hhRz to target any RNA sequence, including the transcript of any gene. The program has clear and valuable advantages over existing hammerhead design services, related to usability, ribozyme quality assessment and multi-objective optimization. It unifies in a single tool the functionalities of targeting ribozyme design software that have been done in the last decade: it finds target sites, computes target accessibility, ribozyme structure validity and potential off-target effects. More importantly for the future community of users, it provides a user friendly interface with the flexibility required for any application: from *in vitro* assays, to gene targeting in *Escherichia coli*, in humans or in any desired species and computes all the complexity of the analysis to simply provide ribozymes that will likely be active over 90% of the time as demonstrated by our *in vitro* assays.

A number of software exist to assist in ribozyme design. Up to now, the best tool in that regard was probably. Aladdin (SeArch computing tooL for hAmmerheaD ribozyme DesIgN) (http://aladdin.ifc.cnr.it/aladdin). This tool, which evolved from an earlier computational method (29,30), is handy and scientifically sound. However, RiboSoft has several advantages: it uses the latest research on target-site accessibility; and it has off-target specificity analysis. Off-target accessibility could be evaluated with RiboSubstrates (31), but the design of ribozymes would then require the use of two different tools, which makes automated design more complicated. RiboSoft also has the advantage of allowing specification of the nature of the environment, the type and size of the ribozyme and the nature of the application. Similarly, the evaluation of possible hhRzs is easier with RiboSoft than Aladdin, because the former provides multi-objective ranking and allows sorting ribozymes according to each parameter, Our experience with these parameters seem to point toward a stronger impact of ‘accessibility 2’ on the ability of a chosen ribozyme to cleave its target.

The other contribution is the design (by RiboSoft) of four hhRz targeting the transcript of the PABPN1 gene, and testing these ribozymes, *in vitro* and *in vivo*, for their cleavage efficiency. A mutated PABPN1 is the root cause of OPMD (oculopharyngeal muscular dystrophy) in humans, a late onset muscle disease associated with progressive ptosis of the eyelids, and dysphagia. The experiments validated the designs as they demonstrated cleavage by each of the four ribozymes. In spite of using single turnover conditions for *in vitro* assays, the results *in vivo* show that the ribozymes designed by RiboSoft lead to an efficient knockdown of PABPN1. This is likely due to the use of a strong promoter for ribozyme expression and higher stability of the structured ribozyme RNAs, as compared to the mRNA, which generally have low expression as compared to most non-coding RNAs. Overall, according to our results, we recommend using two hhRzs for researchers interested in targeting a gene for silencing.

Any upcoming gene targeting projects can benefit from RiboSoft which will easily provide multiple ribozyme designs against any transcript. This fact will allow anyone to select multiple ribozymes in order to attain the best effect possible. Indeed, as we have demonstrated, a dramatic additive effect is obtained by combining two ribozymes, and in the few cases where it would be insufficient, three ribozymes are likely to be enough, making RiboSoft all the more valuable.

There are two ways in which this work can be developed. One direction is that of expanding the computational design tool to include not just hhRz, as it does now, but other ribozymes, such as hairpin and HDV ribozymes. These ribozymes share the basic architecture of a hhRz, which is made of hybridizing arms responsible for the selective capability of the ribozyme and a largely conserved core. As such, many ribozymes can, in principle, be optimized using the same overall multi-objective approach employed by RiboSoft, which essentially takes into account the (i) structure of the ribozyme and exact sequence of its recognition arms; (ii) structure of the region of the targeted cut-site and its exact sequence; (iii) off-target effects, for *in vivo* applications. We cannot see any conceptual or technical impediments to the customization of the general approach to the various ribozymes whose functionality is a function of these three (i–iii) sets of considerations.

In addition, riboswitch-ribozymes have been developed and their use could be greatly extended with proper design tools such as RiboSoft. A riboswitch is an RNA that turns genes on or off, when it binds to a ligand input or a combination of ligand inputs (32). Riboswitches can be combined with ribozymes so that the binding of their ligands trigger RNA cleavage (33,34). Engineered examples of riboswitch-ribozymes implementing Boolean functions include some that respond to small-molecule ligands (14). Allosteric ribozymes, or any other ribozyme, could be added to RiboSoft in the same way as hhRzs have been implemented. In the long run, users could choose from a list of ribozymes with different features to target genes. In principle, any allosteric ribozymes found via SELEX (35) could be implemented for future use, as long as their structures are known. Although it was not packaged as a user friendly software and was designed for self-cleaving ribozymes rather then for ribozymes capable of cleaving any target, recently developed algorithms allowed construction of switchable ribozymes based on the hhRz model and known aptamers (36). This is similar to what has been developed by the group of Ronald Breaker in 2005, where a purely computational approach was developed that found complementary RNA ligands that altered the conformation and hence activity of a hhRz (37). In fact, they tested *in vitro* computationally designed ribozymes that implemented four Boolean logic functions: AND, OR, NOT and YES. It is worth noting that only a precisely delineated part of the ribozyme need to be altered to give the whole ribozyme this unique allosteric capability; the rest of the ribozyme stays fixed. Both of these allosteric ribozymes could be designed by a RiboSoft 2.0.

Another direction for development involves the modification of the design of the resulting hhRz to test their gene silencing functionality, as well as unwanted effects, in living organisms. Selected ribozymes against PABPN1 appear to cleave their target both *in vitro* and *in vivo*, which is promising for future work aiming to replace the mutant transcripts with healthy mRNAs in human cell, thus advancing toward a therapeutic approach to OPMD. Indeed, even when it is theoretically possible to target them, the exact regions harboring mutations are not necessarily ideal cleavage sites. However, addition of a healthy copy with sequence modifications rendering it immune to cleavage, as previously suggested by others (38), allows the use of ribozymes for any type of genes. In fact, even if many deleterious mutations are known for numerous diseases, in such a model for gene therapy it would be possible to target defective genes without even knowing the exact mutations responsible of the disease.

## SUPPLEMENTARY DATA

Supplementary Data are available at NAR Online.

SUPPLEMENTARY DATA
