# Supplementary material for: Automated design of hammerhead ribozymes and validation by targeting the PABPN1 gene transcript
Source: Nucleic Acids Res. 2015 Nov 2;44(4):e39. doi: 10.1093/nar/gkv1111 (PMC4770207; doi:10.1093/nar/gkv1111)
Supplement: SUPPLEMENTARY DATA [file supp_44_4_e39__index.html]

Automated design of hammerhead ribozymes and validation by targeting the PABPN1 gene transcript — SUPPLEMENTARY DATA 

# Automated design of hammerhead ribozymes and validation by targeting the PABPN1 gene transcript

## SUPPLEMENTARY DATA

- SUPPLEMENTARY DATA
