# Supplementary material for: Automated design of hammerhead ribozymes and validation by targeting the PABPN1 gene transcript
Source: Nucleic Acids Res. 2015 Nov 2;44(4):e39. doi: 10.1093/nar/gkv1111 (PMC4770207; doi:10.1093/nar/gkv1111)
Supplement: SUPPLEMENTARY DATA [file supp_gkv1111_nar-02168-met-h-2015-File009.pdf]

Supplementary Information

for the manuscript

Automated Design and In Vitro Validation of Hammerhead Ribozymes Targeting the PABPN1 Gene Transcript

Nawwaf Kharma[1], Luc Varin[2], Aida Abu-Baker[3], Jonathan Ouellet[4,5], Sabrine, Najeh[4], Mohammad-Reza Ehdaeivand[2], Gabriel Belmonte[1], Anas Ambri[1], Guy Rouleau[3] and Jonathan Perreault[4]

[1] Electrical & Computer Eng. Dept., Concordia University, 1455 boul. de Maisonneuve O., Montreal, QC, H3G 1M8, Canada

[2] Biology Department, Concordia University, 7141 rue Sherbrooke O., Montreal, QC, H4B 1R6, Canada

[3] Montreal Neurological Hospital and Institute, 3801 University Street, Montreal, QC, H3A 2B4, Canada.

[4] INRS - Institut Armand-Frappier, 531 boulevard des Prairies, Laval, QC, H7V 1B7, Canada,

To whom correspondence should be addressed:

Phone: 514-8482424 x3117, fax: 514-8482802, e-mail: kharma@ece.concordia.ca or phone: 450-687-5010 x4411, fax: 450-686-5301, e-mail: jonathan.perreault@iaf.inrs.ca

[5] Present address:

Jonathan Ouellet, Department of Chemistry and Physics, Monmouth University, 400 Cedar Avenue, West Long Branch, NJ, 07764

Table of content

Multi-objective optimization and ranking 3
RiboSoft pseudo-code 4
Ribozyme Structure 4
Target Accessibility 4
Specificity Assessment 5
Fitness Evaluation 6
Supplementary methods 7
RFP and 16S Transcripts 7
Additional Ribozyme Transcripts 7
Kinetic measurements of ribozyme cleavage for RFP and 16S 8
Table S1 9
Figure legends 10
Figure S1 11
Figure S2 12
Sequences 13

Multi-objective optimization

The schematic below illustrates the principles of multi-objective optimization with “pareto-optimal” fronts.

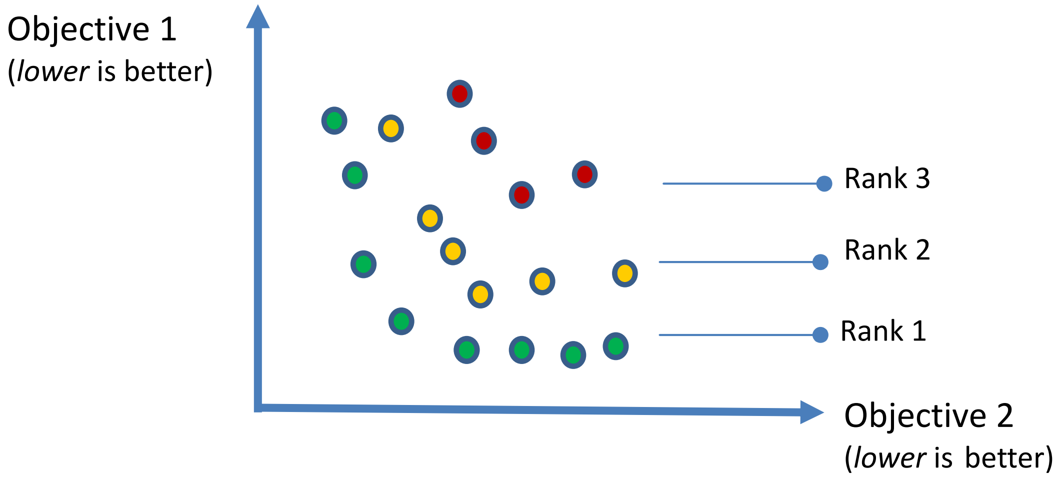

- Ranks of potential solutions (hhRz designs) are based on the concept of domination & non-domination
  - Domination: solution X dominates another solution Y, if (1) Solution X is no worse than Y in all objectives AND (2) solution X is strictly better than Y in at least one objective.
  - Non-dominated solutions: If two solutions are compared, then the solutions are said to be non-dominated with respect to each other if and only if neither solution dominates the other.

- The set of those solutions that are non-dominated by any solution make-up the’ pareto-optimal’ front, and are all assigned the same rank: 1

- If the rank 1 solutions are removed, the next front is comprised of solutions of rank 2

- This layered removal of solution ‘fronts’ is continued until either we don’t need any more solutions or there are simply no solutions left

- The same principles apply independently of the number of objectives (dimensions) assessed

RiboSoft pseudo-code

The following pseudo-code provides brief explanations of the algorithms that evaluate each parameter of the ribozyme candidates.

Ribozyme Structure

```
sum = 0;

for each candidateStructure

  ContinuousPairs = FindContinuousBasePairs();

  for each ContinuousPair in ContinuousPairs

    sum += MeltingTemperature(ContinuousPair);

  end for each;

end for each;
```

A candidateStructure is a possible secondary structure of an hhRz. FindContinousBasePairs will find all double-stranded sequences in the structure, such that there is at most one mismatch in each sequence. This list will be stored in ContinousPairs. For each of these double stranded segments (or ContinuousPair), MeltingTemperature will be computed and added to sum. This sum represents the melting temperature of the whole candidateStructure.

Target Accessibility

Cut-site inaccessibility

```
sum = 0;

for each fold in targetFolds

  ContinuousPairs = FindContinuousBasePairs();

  for each ContinuousPair in ContinuousPairs

    sum += MeltingTemperature(ContinuousPair);

  end for each;

end for each;
```

The algorithm is identical to the one used for Ribozyme Structure explained earlier. Only the regions have changed. The regions are now the cut-site regions in targetFolds.

Disruption energy

```
for each cut-site
  constrainedFolds =
    FoldTargetWithOpenCutsite(target, cut-site);
  cut-site.disruptionE =
    targetFolds.LFE-constrainedFolds.LFE;
end for each;
```

The cut-site is the region of the target substrate that is complementary to the arms of the hhRz. The constrainedFolds represent secondary structures of the target such that the target is forced open at the cut-site. These secondary structures are obtained through the FoldTargetWithOpenCutsites operation. The disruptionE energy is the difference between two energies: targetFolds.LFE and constrainedFolds.LFE. targetFolds.LFE (where "LFE" is "Local Folding Energy"). The former is the free energy of the unconstrained folding of the target substrate, while the latter is the free energy of the target substrate, with the cut-site region forced open.

Specificity Assessment

```
Specificity = 0;
for each cut-site
  results = Queryblast(cut-site);
  for each result in results
    Weight(result);
    if result is XM or XR
      result.Weight = 0;
    end if;
```

```
    Specificity += result.Weight;
  end for each;
end for each;
```

Specificity is initialized to zero. For each cut-site, results will hold information obtained from querying BLAST on that cut-site. A cut-site is defined here as the region of the target that is complementary to the arms of the hhRz. Each BLAST result will be dealt with individually, even if two or more results are attributed to a single transcript. Weight will weigh each result based on the perfection of its match to the hhRz arms. These weighted results will be summed into Specificity. An XM or XR Blast hit will not be used in the specificity calculation.

Fitness Evaluation

```
ParetoRank (candidates, rank)
{
  for each pivot in candidates
    for each candidate in candidates
      if (Dominates (candidate, pivot))
        exit inner-loop,
       continue outer-loop -> try new pivot;
    end for each
    pivot.rank = rank;
  end for each
  remove all x in candidates with pivot.rank = rank;
  ParetoRank(candidates, rank - 1);
}
```

The first time this operation is called, it is called using a list of all the candidates and a rank equal to 1. A pivot is a candidate that will be compared to every other candidate. Candidate A dominates candidate B if it is at least as good as B in all measures of quality

and better than B in at least one quality. If pivot is not dominated by any other as determined by Dominates, then its rank is set to rank. When all candidates have been evaluated, all those that have been assigned the current rank value are removed from the candidates list in this function. The function will be re-invoked with the ParetoRank call, with a decreased set of candidates and a decremented rank value.

Supplementary methods

RFP and 16S Transcripts

The RFP (Red Fluorescent Protein) gene was amplified by PCR using as a template the plasmid BBa_E1010, provided by Biobricks. The PCR reaction was made in one step using as the forward oligonucleotide 5′-TAATACGACTCACTATAGCGTCTAGA-3′ containing the T7 promoter and the reverse oligonucleotide 5′-TTATTAAGCACCGGTGGAG-3′ resulting in a PCR product of 726 nucleotides that was used as the DNA template for transcription. The reaction was made for 35 cycles at an annealing temperature of 51.3°C with the same conditions as other PCR reactions.

Another target RNA was prepared, a region of the 16S rDNA of *Microcystis aeruginosa* NIES 298 was amplified by PCR from genomic DNA using the forward primer 5′-TAATACGACTCACTATAGCGAGAGTTTGATCCTGGCTCAG-3′, containing the T7 so that the template can be used for the transcription, and the reverse primer 5′-GCTTCGGCACGGCTCGGGTCGATA-3′ to generate a DNA template of 822 nucleotides. The same reaction conditions were used at an annealing temperature of 48.9°C for 35 cycles. The size of each DNA template was confirmed using a 2% agarose gel. The DNA was precipitated in ethanol with NaOAc.

In both cases, the RNA was prepared as described for hammerhead ribozymes, except that it was purified on 6% PAGE.

Additional Ribozyme Transcripts

The hhRz designed against RFP and the 16S fragment were all prepared in the same way as the ribozymes against PABPN1 with the following oligonucleotides:

GUC2(4):

TGGGTCGTTTCGTCGCATTTCAGCGACTCATCAGCGTACTTAGAAGGTACCGCTATAGTGAGTCGTATTA

GUC4(1):

CATCCTGTTTCGTCGCATTTCAGCGACTCATCAGCCCGCTTAGTTCCAGCGCTATAGTGAGTCGTATTA

GUC4(6):

ACATCCTGTTTCGTCGCATTTCAGCGACTCATCAGCCCGCTTAAGTTCCGCTATAGTGAGTCGTATTA

GUC7(1):

CGTCCGACGGTTTCGTCGCATTTCAGCGACTCATCAGCGGTTTTAATGCAGAACGCTATAGTGAGTCGTATTA

GUC7(4):

CCGACGGTTTCGTCGCATTTCAGCGACTCATCAGCGGTTTTAATGCAGAAACGCTATAGTGAGTCGTATTA

GUC9(1):

AGACGGTGGTTTCGTCGCATTTCAGCGACTCATCAGACTACTTAGACGCTCGCTATAGTGAGTCGTATTA

GUC9(7):

ACGGTGGTTTCGTCGCATTTCAGCGACTCATCAGACTACTTAGACGCCGCTATAGTGAGTCGTATTA

GUC11(1):

GAAGGTCGTTTCGTCGCATTTCAGCGACTCATCAGACTCCTTAACCGGTGCTCGCTATAGTGAGTCGTATTA

GUC5(m):

GAAACTGTTTTCGTCGCATTTCAGCGACTCATCAGCTTCCTTACGGAATGCCGCTATAGTGAGTCGTATTA

GUC5(3):
TGAAACTGTTTCGTCGCATTTCAGCGACTCATCAGCTTCCTTACGGAAGCGCTATAGTGAGTCGTATTA

GUC3(1):

AGGAAGGTTTCGTCGCATTTCAGCGACTCATCAGTTTGGTTAATTGTCGCTATAGTGAGTCGTATTA

GUC7(5):
AAGTCAGCTTGTTTCGTCGCATTTCAGCGACTCATCAGAAATCTTAAGGTTAACGCTATAGTGAGTCGTATTA

GUC7(m):
AAGTCTGCTGTTTCGTCGCATTTCAGCGACTCATCAGAAATCTTAAGGTTGCTCGCTATAGTGAGTCGTATTA

GUC7(1)(A):
CGTCCGACGGTTTCGTCGCATTTCAGCGACTCATCAGCGGTTTTAATGCAGACGCTATAGTGAGTCGTATTA

GUC2(1)(A):
TGAAGGTCGTTTCGTCGCATTTCAGCGACTCATCAGCGTACTTAGAAGGTCGCTATAGTGAGTCGTATTA

GUC9(1)(A):
AAGACGGTGGTTTCGTCGCATTTCAGCGACTCATCAGACTACTTAGACGCGCTATAGTGAGTCGTATTA

GUC11(5):
GAAGGTCGTTTCGTCGCATTTCAGCGACTCATCAGACTCCTTAACCGGCGCTATAGTGAGTCGTATTA

Kinetic measurements of ribozyme cleavage for RFP and 16S

To monitor the ribozyme cleavage kinetics, 5nM of each ribozyme was added to the cleavage buffer (as described the main text) in the presence of labelled RFP or 16S and in absence of $MgCl_2$. The first step was the incubation at 85°C for 1 minute to allow RNA folding, followed by the immediate transfer on ice for a few minutes. In the second step, 1µl of $MgCl_2$ was added to start the cleavage reaction and this moment was considered as time 0, after which aliquots of 2 µl each were taken at different times. All the rest is the same as described in the main text.

Table S1

Ribozyme (rank)	Tm left	Tm right	Accessibility 1	Accessibility 2	Shape quality	Cleavage	Target
**2(4)**	45.39	35.39	0.38	0.546	0.315	+	RFP
**4(1)**	49.39	29.39	0.519	0.519	0.708	++	RFP
**4(6)**	39.39	31.39	0.624	0.519	0.241	++++	RFP
**5(3)**	40.97	36.97	0.62	0	0.651	-	RFP
**7(1)**	43.39	43.39	0.268	0.227	0.968	++	RFP
**7(4)**	43.29	33.39	0.281	0.227	0.912	++	RFP
**9(1)**	39.39	37.39	0.684	0.226	0.525	++++	RFP
**9(7)**	37.39	31.39	0.684	0.226	0.466	+++	RFP
**11(1)**	52.07	33.39	0.499	0.309	0.536	-/+	RFP
**11(5)**	39.39	35.39	0.648	0.309	0.284	-/+	RFP
**A 2(1)**	39.39	35.39	0.763	0.5	0.976	-	RFP
**A 7(1)**	41.39	43.39	0.635	0.236	0.953	++	RFP
**A 9(4)**	37.39	39.39	0.605	0.212	0.551	+	RFP
**3(1)**	31.39	29.39	0.645	0.652	1	++++	16S
**7(4)**	41.39	33.39	0.338	0	0.653	-	16S
**7(m)**						-	16S
**5(m)**						-	RFP

The table shows the different parameters used by RiboSoft for ranking with the cleavage efficiency for comparison.

Ribozyme(rank) : the numbers like “2(4)” refer to:

> The cut-site position (which is GUC by default), so “2” means the third GUC that exists in the target sequence because the counting begins from 0 (the first GUC is n° 0). The 4 which is in parenthesis is the rank of the ribozyme as determined by RiboSoft (1 being the best).

Tm: melting temperature for one ribozyme arm with the target. Because ribozymes require complementarity to form two stems with the target, both the left and the right Tm are shown.

Accessibility: to be able to hybridize with the template, the ribozyme should find an accessible sequence so the value of the accessibility that goes from 0 to 1 depends on the position of the GUC and the sequence on both sides of it that could be in a stem (so the value is closer to 0) or in a bulge (so the value is closer to 1) so the closer the value is to 1 the better the cleavage is supposed to be.

Shape quality : Each RNA folds in a specific secondary structure, but the hammerhead ribozyme has a consensus structure so the values of the shape quality are the prediction by RiboSoft of the ability for each putative ribozyme sequence to fold in the active ribozyme structure.

Cleavage: those values presented as – and + are the results of testing the ability of the ribozymes on cutting their template. These values reflect both maximum cleavage and speed of cleavage (from time course experiments, data not shown).

-, no detectable cleavage; -/+, ambiguous; +, low; ++, medium; +++, high; ++++, very high.

Note: the “A” written in front of some ribozyme names means “Anterior” and refers to a first set of ribozymes that were tested on RFP.

Figure S1. Some of the ribozymes designed by RiboSoft against RFP RNA were chosen to evaluate the effect of different parameters on the ribozyme activity. Thus, for many sites two ribozymes of different ranks were tested. All ribozymes were designed by RiboSoft (designated by their cut-site number with their ranking in parenthesis) except Rz 5m, which was designed manually (a ribozyme with a catalytic core identical to that used by RiboSoft, but with binding arms chosen manually to include mismatches with the target). Different combinations of 2 or 3 ribozymes were made and the cleavage efficiency was calculated for each reaction.

The 6% polyacrylamide gel shows labelled RFP RNA in the last lane, the higher band corresponds to the full length RFP (726* nt) with no ribozymes. In the following ten lanes, from 2(4) to 5(m), RFP RNA was incubated in presence of one ribozyme in every reaction where, for the ribozymes 2, 4, 7 and 9, RFP was cleaved and we can observe different bands corresponding to the ribozyme cut-sites respectively: 153* nt (site2), 229* nt (site4), 393* nt (site7) and 558* nt (site 9). In the 8th lane, it is not clear whether the ribozyme is active or not because the corresponding cut-site 11 results in an RFP fragment of 702* nucleotides which we cannot differentiate from the full length RFP (although the band at the top does appear to be slightly lower in this lane). The absence of cleavage for the cut-site 5 was expected as the accessibility 2 of the 5(3) is 0 so the ribozyme is not able to hybridize with the target.

The other lanes are combinations of 2 or 3 ribozymes where the full length RFP is not detectable in most cases, with the corresponding bands for cleaved fragments.

Band intensity was measured by using ImageQuant to calculate the cleavage percentage in each case (every ribozyme by itself and the different combinations).

All the ribozymes have a high cutting percentage (88%-98%) except the ribozymes 5(3) and the mismatch control which are not active. The combination of different ribozymes gives better results we can see that all the RNA is degraded when combining three ribozymes (cleavage goes from 97% to 100%). Note that the negative control (RFP alone) is not 0% because of the presence of some background. Also, many weaker bands can be observed likely because the full length RFP RNA was not perfectly pure and may have included a shorter fragment (which could also be cleaved), which is the most likely explanation for these bands, including the bands at the bottom corresponding roughly to a size of 30 nt.

Figure S2. As in Figure S1, three ribozymes were chosen to be tested on a fragment of the 16S RNA of *Microcystis aeruginosa*. The “16S” lane is the RNA in absence of ribozyme, the band represents the labelled target (full length 800*nt). In the other lanes, the same RNA is incubated in presence of ribozymes, with the same convention as explained in Figure S1. The ribozymes 7(m) and 7(4) have an “accessibility 2” of 0 and are not active, as for 5(m) and 5(3) in figure S1.

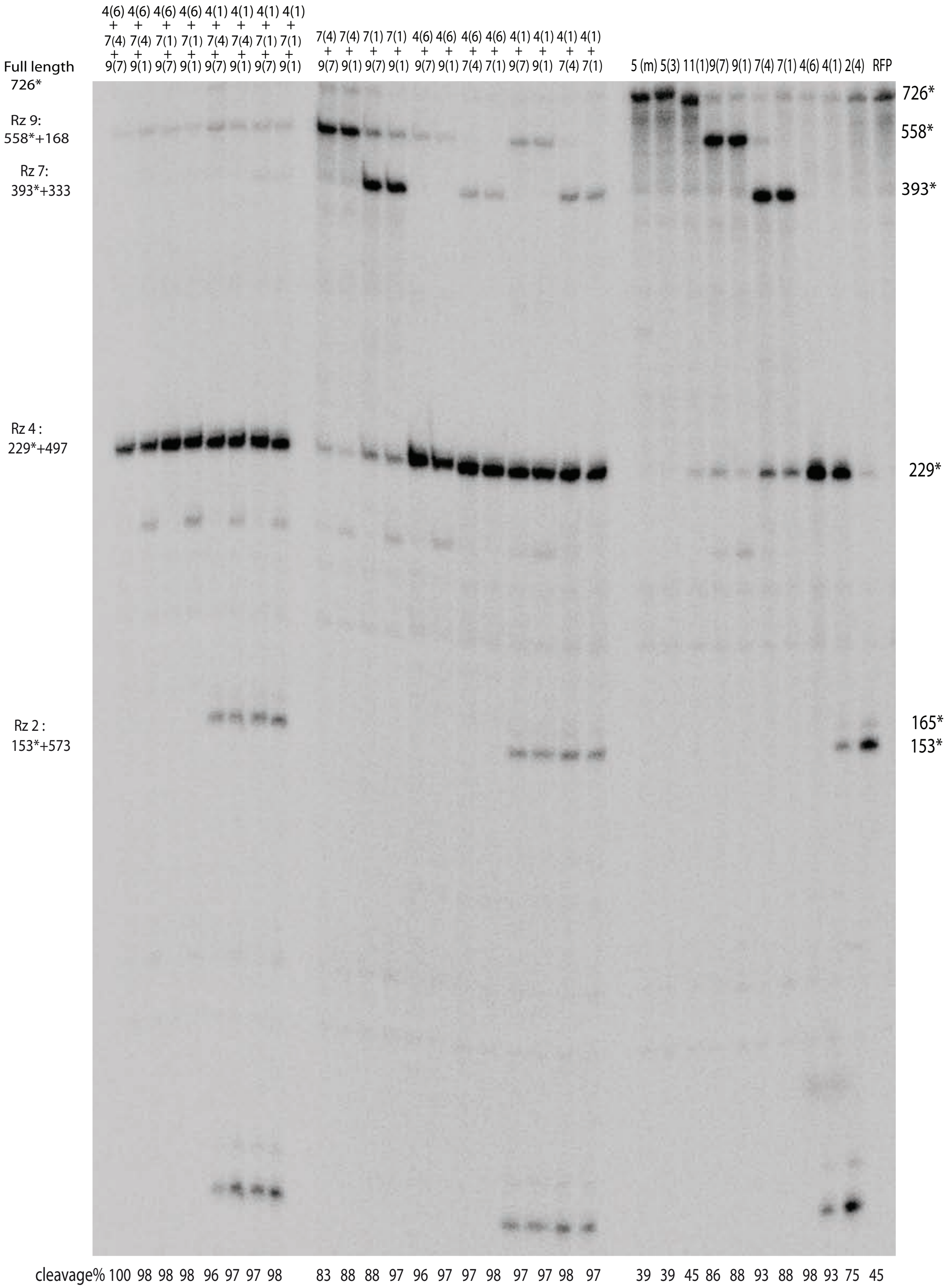

Supplementary Figure S1

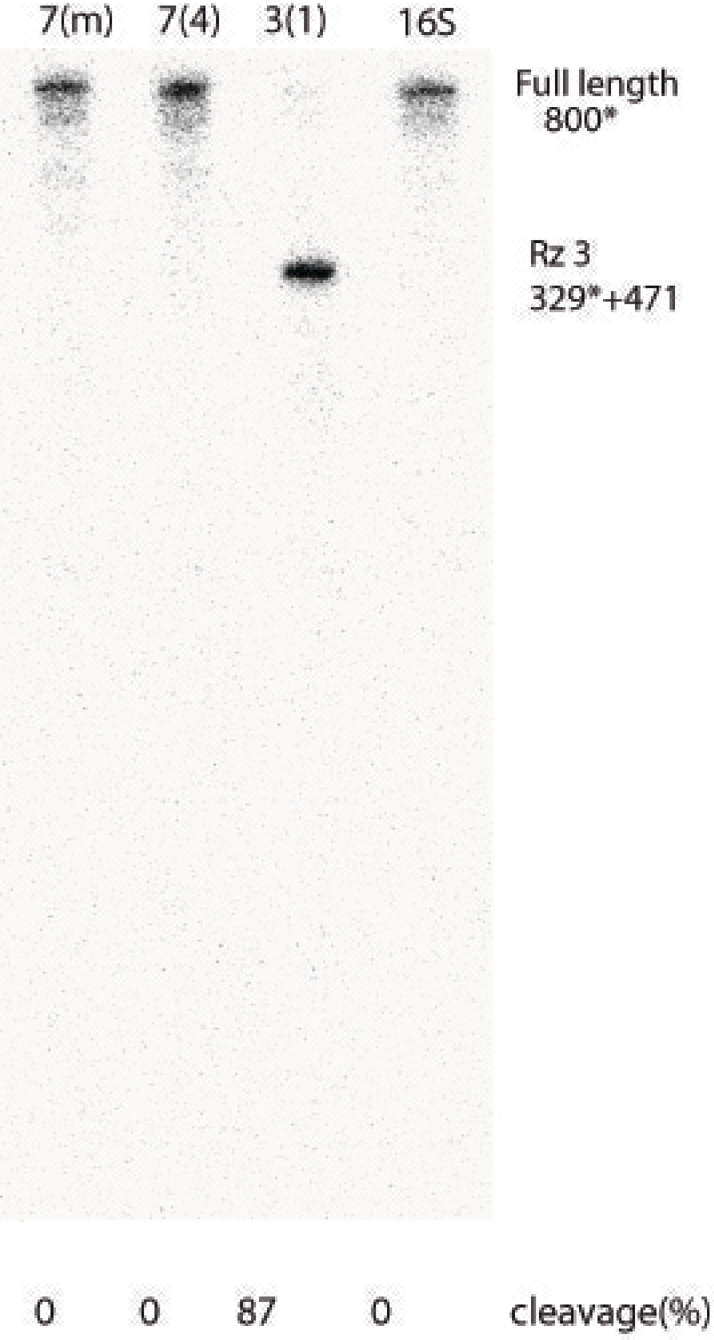

Supplementary Figure S2

Sequences

The target sequences used in this study, as well as the corresponding ribozymes, can be found below. All the potential "GUC" target sites have been annotated in red. Similarly, the different regions of the ribozymes (and corresponding regions on the target RNAs) have been highlighted in different colors to help visualize the ribozymes and cleavage sites: gray, stem 1; green, stem 3; turquoise, stem 2; yellow, conserved regions of CUGANGA and GAAA; finally, pink, nucleotides important for the tertiary interaction between stem I and stem II. For RFP, in cases where regions of complementarity with the ribozymes can vary in length depending on the ribozyme used for targeting, different shadings of the same colors are used.

PABPN1

Transcription product of the new target = 938 nt

144 5′GCGACUACGGGAACGGCCUGGAGUCUGAGGAACUGGAGCCUGAGGAGCUGCUGCUGGAGCCCGAGCCGG
AGCCCGAGCCCGAAGAGGAGCCGCCCCGGCCCCGCGCCCCCCCGGGAGCUCCGGGCCCUGGGCCUGGUUCG
GGAGCCCCCGGCAGCCAAGAGGAGGAGGAGGAGCCGGGACUGGUCGAGGGUGACCCGGGGGACGGCGCCAU
363 UGAGGACCCGGAGCUGGAAGCUAUCAAAGCUCGAGUCAGGGAGAUGGAGGAAGAAGCUGAGAAGCUAAAGG
437 AGCUACAGAACGAGGUAGAGAAGCAGAUGAAUAUGAGUCCACCUCCAGGCAAUGCUGGCCCGGUGAUCAUG
UCCAUUGAGGAGAAGAUGGAGGCUGAUGCCCGUUCCAUCUAUGUUGGCAAUGUGGACUAUGGUGCAACAGC
AGAAGAGCUGGAAGCUCACUUUCAUGGCUGUGGUUCAGUCAACCGUGUUACCAUACUGUGUGACAAAUUUA
GUGGCCAUCCCAAAGGGUUUGCGUAUAUAGAGUUCUCAGACAAAGAGUCAGUGAGGACUUCCUUGGCCUUA
GAUGAGUCCCUAUUUAGAGGAAGGCAAAUCAAGGUGAUCCCAAAACGAACCAACAGACCAGGCAUCAGCAC
AACAGACCGGGGUUUUCCACGAGCCCGCUACCGCGCCCGGACCACCAACUACAACAGCUCCCGCUCUCGAU
867 UCUACAGUGGUUUUAACAGCAGGCCCCGGGGUCGCGUCUACAGGGGCCGGGCUAGAGCGACAUCAUGGUAU
UCCCCUUACUAAAAAAAGUGUGUAUUAGGAGGAGAGAGAGGAAAAAAAGAGGAAAGNARGGAAAAAAAAAA
RAAUUAAAAAAAAAAAAAAGAAAAAACAGAAAAAAAAAAAAAAAUUAAGCGGCCGCCACCGCGGUGGAGCUC
CAGCUUUUGUUCCCUUU 3′

YZ144
5′CAGGCUCCAGUUUAACCUCACUGAUGAGUCGCUGAAAUGCGACGAAACUCCAGGCC 3′

YZ363
5′UCUUCCUCCAUCUAAUCCCUCUGAUGAGUCGCUGAAAUGCGACGAAACUCGAGCUUUGAUA 3′

oldYZ363
5′GGGUCUUUCCUCCAUCUAAUCCCUCUGAUGAGUCGCUGAAAUGCGACGAAACUCGAGCUUUGAUA 3′

Yz437
5′GCAUUGCCUGGUAAAGGUGCUGAUGAGUCGCUGAAAUGCGACGAAACUCAUAUUCAUCU 3′

YZ867
5′CCGGCCCUAACUGUACUGAUGAGUCGCUGAAAUGCGACGAAACGCGACCC **3′**

RFP-Expression Cassette

5’UCUAGAGAAAGAGGAGAAAUACUAGAUGGCUUCCUCCGAAGACGUUAUCAAAGAGUUCAUGCGUUUCA
AAGUUCGUAUGGAAGGUUCCGUUAACGGUCACGAGUUCGAAAUCGAAGGUGAAGGUGAAGGUCGUCCGUA
CGAAGGUACCCAGACCGCUAAACUGAAAGUUACCAAAGGUGGUCCGCUGCCGUUCGCUUGGGACAUCCUG
UCCCCGCAGUUCCAGUACGGUUCCAAAGCUUACGUUAAACACCCGGCUGACAUCCCGGACUACCUGAAAC
UGUCCUUCCCGGAAGGUUUCAAAUGGGAACGUGUUAUGAACUUCGAAGACGGUGGUGUUGUUACCGUUAC
CCAGGACUCCUCCCUGCAAGACGGUGAGUUCAUCUACAAAGUUAAACUGCGUGGUACCAACUUCCCGUCC
GACGGUCCGGUUAUGCAGAAAAAAACCAUGGGUUGGGAAGCUUCCACCGAACGUAUGUACCCGGAAGACG
GUGCUCUGAAAGGUGAAAUCAAAAUGCGUCUGAAACUGAAAGACGGUGGUCACUACGACGCUGAAGUUAA
AACCACCUACAUGGCUAAAAAACCGGUUCAGCUGCCGGGUGCUUACAAAACCGACAUCAAACUGGACAUC
ACCUCCCACAACGAAGACUACACCAUCGUUGAACAGUACGAACGUGCUGAAGGUCGUCACUCCACCGGUG
CUUAAUAA 3’

GUC2(rank4)
5’ GUACCUUCUAAGUACGCUGAUGAGUCGCUGAAAUGCGACGAAACGACCCA3'

GUC4(rank1)
5’CUGGAACUAAGCGGGCUGAUGAGUCGCUGAAAUGCGACGAAACAGGAUG3’

GUC4(rank6)
5’GAACUUAAGCGGGCUGAUGAGUCGCUGAAAUGCGACGAAACAGGAUGU3'

GUC7(rank1)
5’UUCUGCAUUAAAACCGCUGAUGAGUCGCUGAAAUGCGACGAAACCGUCGGACG3'

GUC7(rank4)
5’UUUCUGCAUUAAAACCGCUGAUGAGUCGCUGAAAUGCGACGAAACCGUCGG3'

GUC9(rank1)
5’AGCGUCUAAGUAGUCUGAUGAGUCGCUGAAAUGCGACGAAACCACCGUCU3'

GUC9(rank7)
5’GCGUCUAAGUAGUCUGAUGAGUCGCUGAAAUGCGACGAAACCACCGU3'

GUC11(rank1)
5’AGCACCGGUUAAGGAGUCUGAUGAGUCGCUGAAAUGCGACGAAACGACCUUC3'

GUC5(m)
5’GCAUUCCGUAAGGAAGCUGAUGAGUCGCUGAAAUGCGACGAAAACAGUUUC3’

GUC5(4)
5’CUUCCGUAAGGAAGCUGAUGAGUCGCUGAAAUGCGACGAAACAGUUUCA3’

16S rRNA fragment from *Microcystis aeruginosa*

5’AGAGUUUGAUCCUGGCUCAGGAUGAACGCUGGCGGCGUGCCUAACACAUGCAAGUCGAACGGGAAUCU
UCGGAUUCUAGUGGCGGACGGGUGAGUAACGCGUAAGAAUCUAACUUCAGGACGGGGACAACAGUUGGAA
ACGACUGCUAAUACCCGAUAUGCCGCGAGGUGAAACCUAAUUGGCCUGAAGAAGAGCUUGCGUCUGAUUA
GCUAGUUGGUGGGGUAAGAGCCUACCAAGGCGACGAUCAGUAGCUGGUCUGAGAGGAUGAGCAGCCACAC
UGGGACUGAGACACGGCCCAGACUCCUACGGGAGGCAGCAGUGGGGAAUUUUCCGCAAUGGGCGAAAGCC
UGACGGAGCAACGCCGCGUGAGGGAGGAAGGUCUUUGGAUUGUAAACCUCUUUUCUCAAGGAAGAAGUUC
UGACGGUACUUGAGGAAUCAGCCUCGGCUAACUCCGUGCCAGCAGCCGCGGUAAUACGGGGGAGGCAAGC
GUUAUCCGGAAUUAUUGGGCGUAAAGCGUCCGCAGGUGGUCAGCCAAGUCUGCUGUCAAAUCAGGUUGCU
UAACGACCUAAAGGCGGUGGAAACUGGCAGACUAGAGAGCAGUAGGGGUAGCAGGAAUUCCCAGUGUAGC
GGUGAAAUGCGUAGAGAUUGGGAAGAACAUCGGUGGCGAAAGCGUGCUACUGGGCUGUAUCUGACACUCA
GGGACGAAAGCUAGGGGAGCGAAAGGGAUUAGAUACCCCUGUAGUCCUAGCCGUAAACGAUGGAUACUAG
GCGUGGCUUGUAUCGACCCGAGCCGUGCCGAAGCU 3’

GUC7(m)

5’UUAACCUUAAGAUUUCUGAUGAGUCGCUGAAAUGCGACGAAACAAGCUGACUU3’

GUC7(4)

5’AGCAACCUUAAGAUUUCUGAUGAGUCGCUGAAAUGCGACGAAACAGCAGACUU3’

GUC3(1)

5’ACAAUUAACCAAACUGAUGAGUCGCUGAAAUGCGACGAAACCUUCCU3’
